# Influence of *ACTN3* R577X Polymorphism on Blood Creatine Kinase Levels Relative to Number of Sprints in Brazilian Professional Soccer Players

**DOI:** 10.3390/genes15070896

**Published:** 2024-07-08

**Authors:** Kathleen Y. de Almeida, Hirofumi Zempo, Mika Saito, Tiago Cetolin, Rodrigo dos Santos Guimarães, Andrea Rita Marrero, Aderbal S. Aguiar, Naoki Kikuchi

**Affiliations:** 1 Graduate School of Health and Sport Science, Nippon Sport Science University, Tokyo 158-8085, Japan; kathleen_yasmin@hotmail.com (K.Y.d.A.); sa123ka.v@gmail.com (M.S.); 2 Faculty of Health and Nutrition, Tokyo Seiei College, Tokyo 124-8530, Japan; zempo.hirofumi@gmail.com; 3 Graduate Program in Biochemistry, Federal University of Santa Catarina, Florianópolis 88040-900, Brazil; tcetolin@terra.com.br (T.C.);; 4 Faculty of Sport Sciences, University of Extremadura, 10003 Badajoz, Spain; 5 Graduate Program in Cell and Developmental Biology, Federal University of Santa Catarina, Florianópolis 88040-900, Brazil; andrea.marrero@ufsc.br

**Keywords:** muscle injury, muscle trauma, microinjury, football injury, creatine kinase, creatine phosphokinase, GPS, alpha-actin

## Abstract

This study sought to assess how post-game creatine kinase (CK) levels correlate with the number of sprints and the impact of the *ACTN3* polymorphism on this response. This research constituted a descriptive/observational, retrospective cross-sectional study. DNA was extracted from blood samples for *ACTN3* polymorphism genotyping. CK was measured 48 h after official matches, and the number of sprints (>19 km/h) was tracked using Global Positioning System (GPS) technology. The main cohort included 23 professional soccer players from the top tier of the Brazilian Championship. We analyzed 115 GPS + CK data sets. The replication cohort comprised 18 professional soccer players from the First Division of the Championship, had the same methodology applied, and featured a total of 90 GPS (sprints > 25.2 km/h) + CK data sets. For the main cohort, a significant positive correlation was seen between the number of sprints and the CK levels (*p* = 0.009). Athletes with the *ACTN3* RR genotype had higher CK levels as more sprints were performed during the match (*p* = 0.017). However, the relationship was not found for X allele carriers (*p* > 0.05). For the replication cohort, there was a near-significant correlation between CK levels and the number of sprints (*p* = 0.05), and RR individuals showed a significant association (*p* = 0.01), whereas X allele carriers did not (*p* = 0.06). A greater number of sprints during matches is linked to higher CK levels, primarily among players with the *ACTN3* RR genotype, which is potentially due to an increased presence of type II muscle fibers. These findings were replicated for both cohorts of elite Brazilian soccer players, emphasizing the importance of genetic factors in injury prevention.

## 1. Introduction

Monitoring the fatigue after training and matches in soccer is a common practice as a result of the growing physical demands for elite players. Top-tier soccer players engage in an exhaustive routine, with approximately 60 matches over the course of a season, averaging around 5.5 matches per month [1]. Studies interested in analyzing time-motion in soccer have used Global Positioning System (GPS) to measure external load during training and matches [2,3,4,5]. The Premier League has seen players reaching higher maximum velocities, indicating more frequent bursts of intense acceleration, deceleration, and changes in direction during matches [1], which are factors that are crucial for success in professional soccer [6,7,8]. However, these high demands in soccer practice can result in a decrease in physical performance that can last for several hours or even days as a result of residual and acute fatigue [9]. The extent of this fatigue increases in the first 24 h following a match and reaches its maximum level between 24 and 48 h post-match [4,9]. 

To evaluate the degree of muscle damage caused by soccer matches, several studies have utilized pre-match creatine kinase (CK) levels and post-match up to 120 h later [10], but the levels are suggested to peak between 14 and 48 h post-match [11]. CK is an enzyme involved in the energy production in muscles and is released in the blood in the occurrence of cellular microinjuries. Studies have found that high intensity and sprint distance, as well as sprint number, during a game are correlated with CK changes after the match [11]. The most sensitive external load parameter to predict CK changes 24 h after a match is metrics related to high-intensity running (>19.8 km/h) [12], such as sprints frequency. 

The genetic factor has been recognized as a new approach to identify the susceptibility, severity, and recovery time of non-contact musculoskeletal soft-tissue injuries (NCMSTIs), which account for more than 50% of injuries in sports [13]. In 2009, Collins and Raleigh suggested that genetics affects acute soft-tissue injuries because many genes have a direct impact on the function and structure of these tissues [14]. Several studies on single nucleotide polymorphisms (SNPs) in genes have shown that different genotypes have varying soft tissue structure and function capacities, leading to football players having different injury incidence, severity, and recovery time [15,16,17].

The *ACTN3* R577X is a single-nucleotide polymorphism (SNP) where the mutated allele is characterized by a change in one cytosine for a thymine. This substitution leads to a premature stop codon instead of the amino acid arginine in the α-actin-3 protein located at the Z line of the sarcomere [18]. The wild-type allele, called R, is associated in previous studies with higher strength and power, and the mutate allele, called X, is linked to better muscle endurance performance [19,20]. The *ACTN3* polymorphism can affect markers of muscle damage and the inflammatory response associated with exercise and sports, as stated by Baumert et al. (2016) and Pimenta et al. (2012) [21,22]. The XX genotype in players has been found to increase their susceptibility to non-contact musculoskeletal injuries in multiple studies [13,23], and players with this genotype require more time to recover and return to play after experiencing such injuries [17]. However, populational differences, the fact that only a small number of the studies have investigated soccer, the lack of study designs that examine real-life match situations, restricted access to top-level professional teams, and the fact that most studies so far were performed in European athletes indicate that this association still needs to be further investigated [24].

Therefore, this study aimed to evaluate the relationship between post-game creatine kinase (CK) levels and the number of sprints as a match load parameter, during the competitive season. Moreover, the study also aims to consider the influence of the *ACTN3* gene polymorphism on this response in high-level Brazilian professional soccer players. Given the influence of the ACTN3 gene polymorphism on muscle endurance and recovery dynamics in sports, we hypothesize that Brazilian professional soccer players with the XX genotype exhibit higher post-game creatine kinase (CK) levels compared to those with the RR or RX genotypes, and CK levels reflect the intensity of muscle damage and recovery demands in elite soccer players.

## 2. Materials and Methods

### 2.1. Cohort and Data Collection

This research was a descriptive/observational, retrospective cross-sectional study. The number of athletes was calculated based on the number of players active in the Brazilian Series A football championship over the past 5 years, considering an expected confidence interval of 90%, but taking into account the maximum resource availability and logistical feasibility of the study. The sampling process was intentionally non-probabilistic. Participants are professional athletes recruited through established contacts with club technical staff, who were informed in advance about the study’s characteristics, objectives, and procedures, and expressed their consent and interest in participating. Due to the inherent limitations in sample size, the study design also included the consideration of replicability across different teams rather than relying solely on data from a single team. This approach was intended to enhance the robustness and generalizability of the findings.

#### 2.1.1. Main Cohort

The main cohort comprised 23 professional soccer players (25 ± 3.9 years old; 77 ± 5.6 kg; 180 ± 4.7 cm) belonging to first division of the Brazilian Championship. The athletes were required to meet specific inclusion and exclusion criteria to participate in the study. The inclusion criteria required the participants to be Brazilian male professional soccer athletes from professional soccer clubs in the First Division of the Brazilian Soccer League with a minimum of two uninterrupted years of sports practice before the beginning of this study. However, athletes who had experienced bone and/or muscle injuries in the three months prior to the beginning of the study or who did not meet the inclusion criteria were excluded.

On average, these athletes engaged in training for approximately 6–7 days a week, participating in around 6–8 training sessions that typically lasted for about 70 min each. This training regimen was complemented by weekly soccer matches, each lasting around 90 min.

The number of sprints during each competition was estimated with Global Positioning System (GPS) (VectorS7, Catapult Innovations, Melbourne, Australia), which was worn on the chest during all the game time. Sprints were considered as >19 km/h. The data were collected in the end of the season accordingly to the number of times the player participated in official games for more than 45 min, during the seasons of 2018 and 2019. In order to standardize the number of measures and avoid the outliers, given that CK levels can have a high level of fluctuation, five median CK measures were used together with their corresponding GPS data for each athlete who participated for more than 60 min per game, totaling 115 combinations of GPS + CK data analyzed.

The post-game CK level was measured 48 h after official games of the Brazilian Championship. The measurement of CK was performed by taking capillary blood from the digital pulp using an automatic lancet device. In this method, capillary blood was drawn into a heparinized capillary tube (Cat no 955053202 Reflotron) and pipetted onto a CK reagent strip (Cat no 1126695 Reflotron), which was subsequently inserted into the Reflotron Analyzer (Boehringer-Mannheim, Mannheim, Germany).

The study was approved by the Ethics Committee on Human Research at the Federal University of Santa Catarina under number 3,621,353/2019. In order to participate in the study, the athletes had to provide their voluntary consent and were fully informed about the study’s purpose and procedures. The research was conducted in accordance with Resolution 466/2012 of the Brazilian National Health Council.

#### 2.1.2. Replication Cohort

The replication cohort consisted of 18 professional soccer players (29 ± 4.8 years old; 75 ± 12 kg; 178 ± 8.4 cm) who are members of a soccer team from the First Division of the Brazilian Soccer League. The replication cohort consists of different footballers and teams not included in the main cohort. The exclusion and inclusion criteria were the same as the main cohort, just as the CK and GPS (VectorS7, Catapult Innovations, Melbourne, Australia) measurement processes, which were carried out in the season of 2023. However, for the replication cohort, the sprint velocity was considered as >25.2 km/h for the purpose of setting a mechanical threshold. For the replication cohort, there was a total of 90 GPS + CK data analyzed. 

Similarly to the main cohort, on average, these athletes engaged in training for approximately 6 days a week, participating in around 6 training sessions that typically lasted for about 75 min each. This training regimen was complemented by one weekly soccer match, lasting at least 90 min.

### 2.2. DNA Analysis 

#### 2.2.1. Main Cohort

At the start of the season, 4 mL of blood samples was collected from the athletes using BDVacutainer^®^ tubes by one of the investigators that is trained for this type of procedure. The DNA was extracted from the leukocyte layer using a salting-out protocol involving two lysis solutions, SDS, sodium perchlorate, isopropyl alcohol, and ethanol. The extracted DNA was incubated at 56 °C in a water bath and then stored in a refrigerator at −20 °C. The purity of the DNA samples was measured using a Nanovue Plus^®^ spectrophotometer and diluted to 50 ng of DNA per μL. To genotype the *ACTN3* R577X polymorphism, a PCR was performed using specific primers, and the resulting product was digested with Ddel enzyme. The fragments were then separated on a 3% agarose gel using electrophoresis. The adapted protocol from Mills et al. was used [25].

#### 2.2.2. Replication Cohort

Saliva samples were gathered using an Oragene DNA self-collection kit (DNA Genotek, located in Kanata, ON, Canada) in accordance with the manufacturer’s instructions. The *ACTN3* R577X (rs1815739) polymorphism was determined using the TaqMan SNP genotyping assay (Assay ID: C____590093_1_) and the Real-Time PCR System (CFD-3120J1; Bio-Red, Hercules, CA, USA). The TaqMan assays for genotype assessments were analyzed using CFX Manager Software version 2.1 from Bio-Rad.

### 2.3. Statistical Analysis 

Statistical analyses were conducted using SPSS statistical package version 25.0 for Mac (SPSS Inc., Chicago, IL, USA). The Hardy–Weinberg equilibrium for both genotypes was assessed using Pearson’s χ^2^ test. To compare groups, we employed the χ^2^ test and Fisher’s exact test. Linear Logistic regression was performed to verify the association between the sprint number and CK levels in the different genotypes. The *p*-values < 0.05 were considered statistically significant. Graphs were made with ggplot2 package R (version 4.1.3). 

## 3. Results

Both cohorts did not deviate from the Hardy–Weinberg equilibrium (main cohort *p* = 0.99, replication cohort *p* = 0.95). The subjects’ characteristics are presented in Table 1. The athletes from the replication cohort presented higher age but lower CK (U/L) and sprints number mean compared to the main cohort (Table 1). Out of the 23 players from the main cohort, 13 were RR genotype and 10 were X allele carriers (RX and XX genotypes). Athletes with the *ACTN3* RR genotype had significantly higher mean weight (*p* < 0.001) and height (*p* = 0.02), while X athletes were significantly older (*p* = 0.04). For each player, there was an average of 10 ± 4.8 measures of sprints determined by GPS combined with the corresponding CK levels of that game. The RR athletes had significantly higher mean CK levels compared to X individuals (*p* < 0.001), but there was no significant difference in the number of sprints between the genotypes (*p* = 0.2) (Table 1). In Table 2, other game variables are also presented for each cohort.

**Figure 1:**
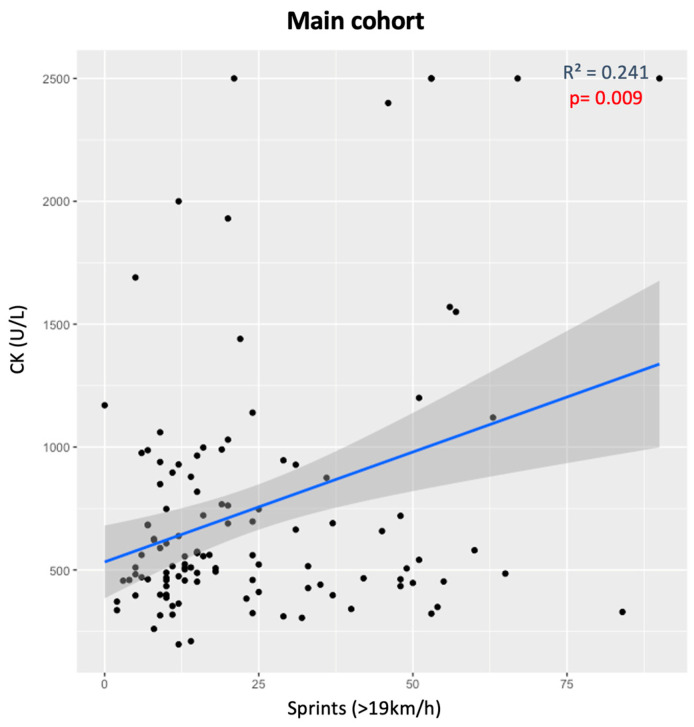
CK levels and number of sprints (>19 km/h) for all athletes in the main cohort.

**Figure 2:**
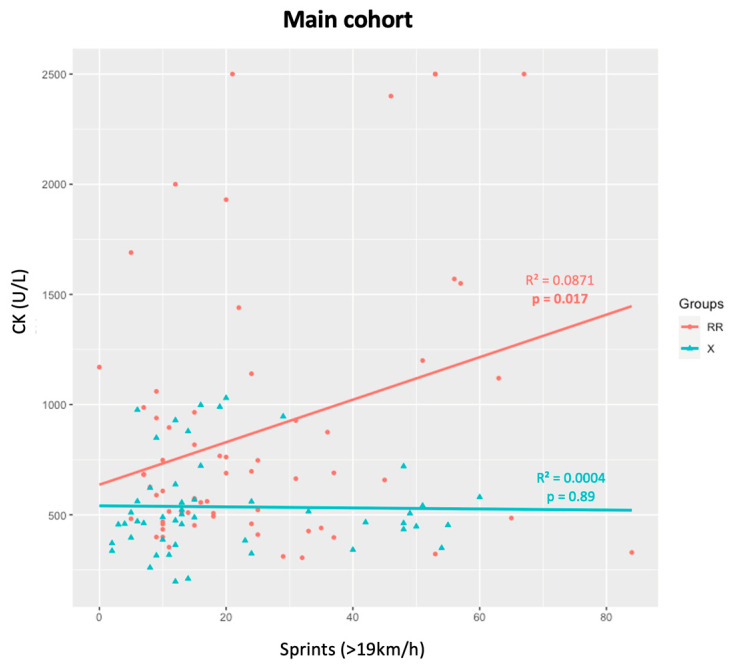
CK levels and number of sprints for RR and X athletes in the main cohort.

In the main cohort of athletes who played more than 60 min, five median CK measures and corresponding GPS data were standardized and analyzed. A significant positive correlation was seen between the number of sprints (>19 km/h) performed during the game and the CK levels (*p* = 0.009) (Figure 1). When divided by the *ACTN3* genotypes, athletes with the RR genotype had higher CK levels as more sprints were performed (*p* = 0.017). However, X allele carriers did not present any significant relationships (*p* > 0.05) (Figure 2).

For the replication cohort, eight individuals had the RR genotype and ten individuals were X allele carriers. There was no difference in age, height, and weight, but RR individuals had significantly higher CK levels (*p* = 0.005), and X allele carriers performed more sprints over 25.2 km/h (*p* = 0.02). For each player, there was an average of 14 ± 5.3 measures of GPS combined with its respective CK levels of that game.

**Figure 3:**
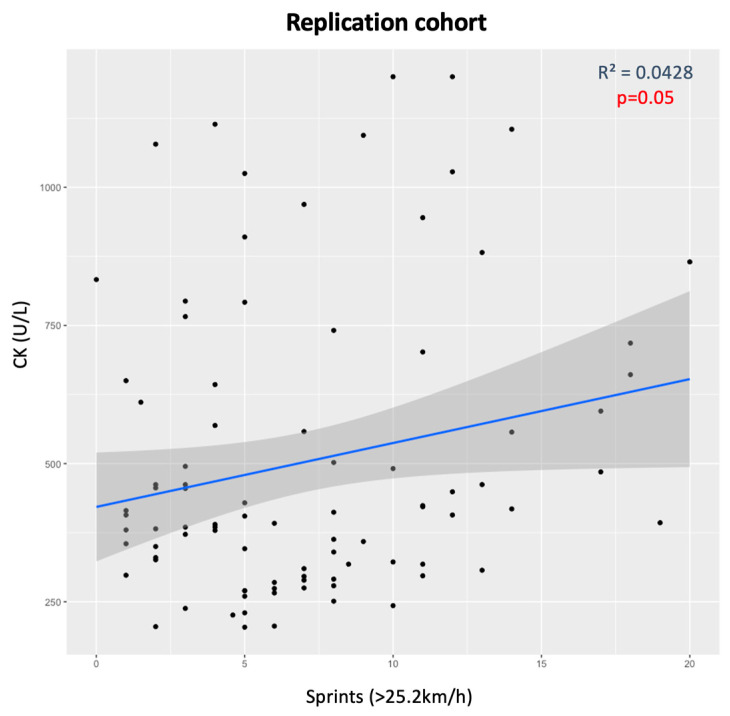
CK levels and number of sprints (>25.2 km/h) for all athletes in the replication cohort.

**Figure 4:**
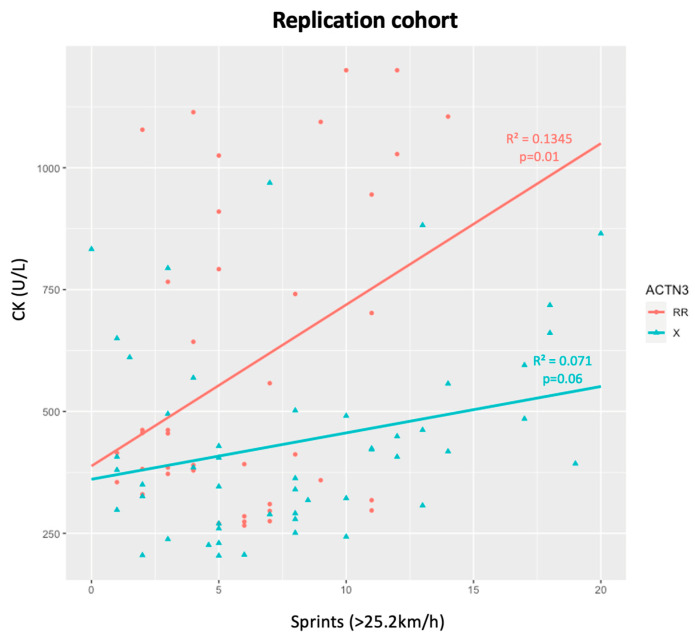
CK levels and number of sprints for RR and X athletes in the replication cohort.

There was an almost statistically significant trend of association between CK levels and the number of sprints in the replication cohort (*p* = 0.05), as the more sprints that are performed (>25.2 km/h), the higher the CK levels (Figure 3). When dividing by genotypes, RR individuals showed the same association (*p* = 0.01), while X allele carriers only presented a trend that was not significant (*p* = 0.06) (Figure 4). 

## 4. Discussion

In this study, we observed that the greater the number of sprints (>19 km/h) performed during the game, the higher the concentration of the biomarker creatine kinase in the blood. However, contrary to our expectations, when dividing the subjects according to the *ACTN3* R577X polymorphism, this relationship was significant for individuals with the *ACTN3* RR genotype, whereas the X allele was not associated with an increase in CK levels associated with sprinting. In addition, RR genotype carriers also presented higher CK levels compared to X allele carriers. These associations initially observed in the main cohort were subsequently confirmed by our replication cohort, which considered sprinting in a higher velocity of >23 km/h.

Creatine kinase (CK) is an intracellular protein that has a peak level ranging from 70% to 250% of baseline within 24 to 48 h aftermath. Analysis of soccer revealed that it can take anywhere between 24 and 120 h for metabolic disruptions to return to their normal state [26]. This response can be mainly attributed to the high-intensity components of the match [27,28], which is a heavy influence for the outcome of a soccer game [6].

In soccer, a study carried out by Pimenta and collaborators (2012) showed that the CK levels after acute training were generally higher for *ACTN3* XX professional Brazilian players 4 h after exercise. However, the results also showed that the R allele was significantly associated with higher levels of interleukin-6 (IL-6), which is a biomarker for the inflammatory response [22]. In contrast, Coelho et al. found that RR presented higher levels of CK both before and 4 h after the soccer game in U-16 category players. What was hypothesized by the authors is that in the context of self-regulated activities, such as soccer, compared to standardized work tasks, it is highly probable that individuals with the *ACTN3*-RR/RX genotype would exhibit superior performance in activities that demand strength and power. Consequently, this could lead to increased levels of post-microtrauma muscle activity in RR/RX individuals [29].

Interestingly, the aforementioned study was also conducted on Brazilian athletes, just like our study. Likewise, de Lima and collaborators (2021), in a cohort of Brazilian individuals after downhill running, saw an increase in serum creatine kinase (CK) activity in both RR genotype and X allele carriers both at two and four days after exercise. Notably, the RR group had more pronounced increases in CK activity than those with the X allele. This corresponds with the greater declines in isometric peak torque (IPT) witnessed in the RR group during the same study, indicating that these individuals suffered more substantial exercise-induced muscle damage (EIMD) [30]. Pereira et al. (2022) observed the same relationship in healthy male individuals, also from Brazil, where the R allele was significantly (*p* < 0.01) associated with greater increases in CK, myoglobin, and cortisol after acute strength training sessions [31]. It is known that there are population differences when it comes to the influence of the *ACTN3* R577X polymorphism. The fact that our study presented the same results in the main and replication cohorts supports the idea that for the Brazilian population, the effect of *ACNT3* R577X on CK levels as an indicator of microinjury may be different from studies in other populations.

Even with higher CK levels, the RR genotype did not show a higher number of sprints compared to X allele carriers. The reason for the results can be explained by the fact that high-intensity sprinting can selectively damage type II fibers, leading to a reduction in peak power output (PPO) [27]. The *ACTN3* polymorphism was previously related to the amount of each muscle fiber type, where the RR genotype was significantly associated with a higher concentration of type II muscle fibers [32]. Studies involving human and animals have showed that type II muscle fibers, especially type IIb, experience more damage following eccentric exercise compared to type I fibers [33,34]. The reasons for that can be the reduced oxidative capacity, the preferential activation of fast-twitch muscle fibers when eccentric contractions occur, the regulation of calcium homeostasis, structural differences such as for example the narrower Z-line in fast-twitch fibers (type II) compared to slow-twitch fibers (type I), and the composition of the fibers with specific proteins and thin filaments [35,36,37]. The fast fibers are potentially an area of higher stress levels transmission due to their lower molecular weight and reduced elasticity titin isoform [38,39], and besides that, the fact that fast fibers present smaller isoforms of myomesin and nebulin can result in greater stress in the cytoskeleton [36,38].

Additionally, Venckunas et al. (2012) offered an alternative explanation for the more significant exercise-induced muscle damage (EIMD) observed in RR individuals, which was also evident in their study. The authors suggested that X allele carriers might initially be more susceptible to EIMD. However, this susceptibility could trigger a subsequent “repeated bout effect” resulting in an adaptive response that ultimately renders X allele carriers more resistant to EIMD. In other words, their initial vulnerability to muscle damage may lead to a phenotypical protective response due to the repeated bout effect [40].

It is important to mention that our previous study also investigated the association of the same polymorphism with the incidence and severity of muscle injuries in Brazilian soccer athletes. The study indicated that the X allele is not only associated with a higher incidence of injuries per season but also plays a big role in predicting the injury severity, which is measured as the number of days of absence as a result of the suffered injury. In the study, individuals with the XX genotype were more likely to suffer from severe injuries compared to the RR and RX genotypes (OR: 5.141, 95% CI: 1.472–17.961, *p* = 0.010) [17]. However, the study took into consideration only the injuries that lead to some kind of medical condition with at least 1 day of absence due to the injury. This is a different situation compared to biomarkers such as the CK levels, which can suggest the extension of injuries at a cellular level, and can be associated with a future medical occurrence or not.

In practical terms, the findings from this study can be added to the literature of the use of genetic and biomarker information to guide the development of personalized training programs and injury prevention strategies for professional soccer athletes. By taking into account individual genetic profiles and biomarker responses such as CK levels, coaches and sports scientists can customize training intensities and recovery protocols, enhancing athletic performance while reducing the risk of injury.

However, the limitations of this study should be acknowledged to interpret the findings. One significant limitation of this study is the absence of detailed information regarding the race or ethnicity of the participants. As race can play a significant role in genetic polymorphisms and susceptibility to certain conditions, the absence of these data prevents a comprehensive understanding of potential variations within the study population. In addition, this study primarily focused on measuring creatine kinase (CK) levels as a marker of muscle injury. While CK is a widely accepted biomarker, it is not the sole indicator of muscle damage. Future research should consider incorporating a broader array of biomarkers and assessments to provide a more comprehensive understanding of muscle injury and recovery in soccer players, and it could also consider some factors that can interfere in this response, such as diet and supplementation. Another limitation of our study is the variation in thresholds in sprint velocity defined by each soccer team (both main and replication cohort). This diversity may potentially affect the generalizability of our findings. Finally, it is known that different positions in football can entail different physical demands and physiological characteristics. Having a cohort large enough to have good representation for each position is important to understand positional particularities and possible different types of responses within the sport. Therefore, while this study contributes valuable insights into the relationship between *ACTN3* R577X, sprint performance, and muscle injury in high-level professional soccer players, these limitations should be acknowledged when interpreting the results. Future research endeavors should aim to address these limitations by expanding the scope of biomarker assessments, collecting more comprehensive demographic data to enhance the understanding of muscle injury, accounting for positional differences, and considering a more diverse range of genetic factors. Also, it would be interesting if future studies could recruit a larger cohort of professional soccer athletes, although there is an inherent difficulty regarding the access to players within this type of elite cohort and access to real game conditions.

## 5. Conclusions

The CK levels are significantly related to the number of sprints performed during the soccer match; the more sprints that are performed, the higher the CK levels. However, this CK response to sprinting differs depending on the *ACTN3* R577X polymorphism, since this relationship was only observed for *ACTN3* RR genotype players but not for X allele carries. In addition, the RR genotype showed higher CK levels compared to the X allele. This association can be explained by the fact that the RR genotype is associated with a higher proportion of type II muscle fibers, a higher risk of microinjuries, and also a possible repeated bout effect. The findings indicate that taking genetic factors into account is essential for injury prevention and, consequently, enhancing sports recovery and rehabilitation.

## Figures and Tables

**Table 1:** Subjects’ characteristics divided in each cohort (presented as mean ± SD).

	Main Cohort			Replication Cohort		
	All	RR	X	All	RR	X
n	23	8	10	18	8	10
Age (years)	25 ± 3.9 **	28 ± 4.2 *	30 ± 5.4 *	29 ± 4.8 **	28 ± 4.2	30 ± 5.4
Height (cm)	180 ± 4.7	180 ± 8.8 *	175.8 ± 7.9 *	178 ± 8.4	180 ± 8.8	175.8 ± 7.9
Weight (kg)	77 ± 5.6	76 ± 8.3 *	77 ± 10.6 *	75 ± 12	76 ± 8.3	77 ± 10.6
CK (U/L)	728 ± 500 **	876 ± 598 *	536 ± 211 *	503.5 ± 263 **	587.4 ± 311.6 *	436.4 ± 191.3 *
Sprints (n)	22.9 ± 17.7 **	24.8 ± 18.3	20.6 ± 16.8	7 ± 4.7 **	6 ± 3.4 *	7.9 ± 5.3 *

* Comparison between the genotypes where the *p*-value was under 0.05. ** Comparison between main and replication cohort in all subjects where the *p*-value was under 0.05.

**Table 2:** Other match variables for each cohort (presented as mean ± SD).

	Main Cohort	Replication Cohort
Total Distance (m)	6709 ± 3147	7312 ± 3129
Maximum Velocity (km/h)	28.1 ± 3.5	29 ± 2.0
HRmax (bpm)	186.9 ± 19.9	173 ± 41.4

## Data Availability

The data from this study can be obtained from the corresponding author upon making a reasonable request.
